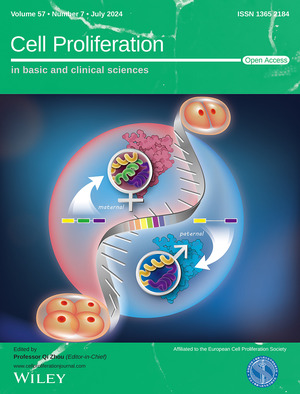# Featured Cover

**DOI:** 10.1111/cpr.13709

**Published:** 2024-07-01

**Authors:** Fan Li, Najmeh Karimi, Siqi Wang, Tianshi Pan, Jingxi Dong, Xin Wang, Sinan Ma, Qingtong Shan, Chao Liu, Ying Zhang, Wei Li, Guihai Feng

## Abstract

The cover image is based on the Letter to the Editor *mRNA isoform switches during mouse zygotic genome activation* by Fan Li et al., https://doi.org/10.1111/cpr.13655.